# Optimal allocation of the limited oral cholera vaccine supply between endemic and epidemic settings

**DOI:** 10.1098/rsif.2015.0703

**Published:** 2015-10-06

**Authors:** Sean M. Moore, Justin Lessler

**Affiliations:** Department of Epidemiology, Johns Hopkins Bloomberg School of Public Health, Baltimore, MD 21205, USA

**Keywords:** cholera, vaccine, vaccine stockpile, minimax, reactive vaccination

## Abstract

The World Health Organization (WHO) recently established a global stockpile of oral cholera vaccine (OCV) to be preferentially used in epidemic response (reactive campaigns) with any vaccine remaining after 1 year allocated to endemic settings. Hence, the number of cholera cases or deaths prevented in an endemic setting represents the minimum utility of these doses, and the optimal risk-averse response to any reactive vaccination request (i.e. the minimax strategy) is one that allocates the remaining doses between the requested epidemic response and endemic use in order to ensure that at least this minimum utility is achieved. Using mathematical models, we find that the best minimax strategy is to allocate the majority of doses to reactive campaigns, unless the request came late in the targeted epidemic. As vaccine supplies dwindle, the case for reactive use of the remaining doses grows stronger. Our analysis provides a lower bound for the amount of OCV to keep in reserve when responding to any request. These results provide a strategic context for the fulfilment of requests to the stockpile, and define allocation strategies that minimize the number of OCV doses that are allocated to suboptimal situations.

## 1. Introduction

There are an estimated 2.8 million cases and 91 000 deaths owing to cholera in endemic areas annually [1]. In addition, large epidemics can occur in non-endemic regions, such as the epidemic that has infected over 720 000 and killed nearly 9000 in Haiti, Dominican Republic and Cuba since it began in October 2010 [2]. Cholera prevention and control efforts traditionally focus on providing clean drinking water, adequate sanitation and proper food hygiene [3]. However, infrastructure improvements are costly and often difficult to implement quickly or efficiently. The recent international licensure of two low-cost oral cholera vaccines (OCVs; Dukoral^®^ and Shanchol^®^, has renewed interest in using vaccination for cholera control. In 2009, the World Health Organization (WHO) Strategic Advisory Group of Experts (SAGE) for the first time recommended the use of reactive vaccination as an outbreak control strategy [4], and in 2013, a global stockpile with a target size of 2 million OCV doses was established to help control cholera epidemics [5,6]. The Gavi Vaccine Alliance recently approved a contribution for the expansion and broader use of the global OCV stockpile until 2018 [7]. Despite these developments, the global OCV production capacity is expected to be inadequate to meet potential demand for the foreseeable future [1].

The global OCV stockpile aims to quickly provide doses to countries who wish to use it in reactive vaccination campaigns in response to epidemics or for pre-emptive use in humanitarian emergencies. These requests are prioritized over proactive OCV use in endemic settings, because the cumulative incidence in large epidemics such as those recently experienced in Haiti and Zimbabwe is higher than the annual incidence in endemic regions [8,9]. The case fatality rate (CFR) is often higher during epidemics as well, owing to the lack of established capacity for treating cholera [10]. Reactive vaccination also avoids the uncertainties inherent in proactive vaccination, because vaccine is not allocated until after the outbreak has started. However, timing is crucial for a reactive vaccination campaign and delays in vaccine deployment will reduce the number of cases averted [11,12].

When the OCV stockpile receives a request for reactive deployment, it must decide if fulfilling it maximizes the public health benefit of the stockpile, or if doses would be better held in reserve to be used in future requests; a difficult decision given that it is unknown if such future requests will occur. By the guidelines of the stockpile, OCV doses will be available on a rotating basis and any doses nearing the end of their shelf-life can be allocated to cholera endemic settings before expiration. Hence, the number of cholera cases these doses could prevent in an endemic setting represent the minimum utility of the requested OCV doses if they are held in reserve instead of allocated immediately. For any particular stockpile request, the expected number of cases that would be prevented if the request is approved can therefore be compared with the utility of the same number of OCV doses in an endemic setting. The most risk-averse decision in this scenario is one that would minimize the possible combined loss (in terms of the number of vaccine-preventable cholera cases that occur), which in this scenario is equivalent to maximizing the minimum utility of the OCV doses. This approach is referred to as a minimax strategy because it optimizes the minimum possible utility of the current amount of vaccine on hand, by allocating doses in such a way that their utility would be maximized under the worst possible request scenario for their future use [13]. This strategy comes from game theory, where it represents the best risk-averse strategy in a zero sum game against an active opponent [14]. While minimax is not the globally optimal strategy for allocation of OCV (because it does not consider potential future stockpile requests that could prevent more cases), it minimizes the potential loss in utility of vaccine incurred by holding the doses in reserve.

**Figure 1.**
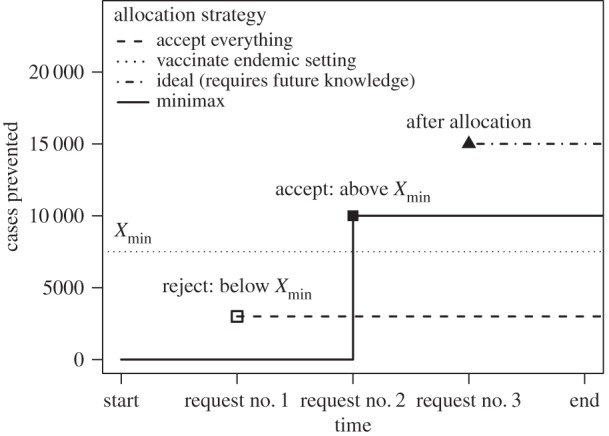
The number of cholera cases prevented via vaccination with a given set of oral cholera vaccine (OCV) doses when using a minimax strategy compared with several other simple strategies (accept all requests, vaccinate proactively in an endemic setting or accept optimal request assuming perfect knowledge of all future requests). For the minimax strategy (solid line) the decision to fulfil a request is made by comparing the expected cases prevented by the request to the number of cases that would be prevented in an endemic setting (*X*_min_ = 7500), which represents the minimum utility of the OCV doses. It is assumed that three requests for vaccine are received in chronological order: the first request will only prevent 3000 cases (open square) so it is rejected, the second request will prevent 10 000 cases and is accepted (solid square), and the third request cannot be accepted because all OCV doses have already been allocated.

The goal of this study was to find an optimal allocation between reactive vaccination and holding OCV doses in reserve under a variety of scenarios. Figure 1 presents a simple example portraying how the minimax strategy maximizes the minimum utility of the OCV stockpile for each stockpile request. The minimum utility of the existing OCV doses in the stockpile is the number of cholera cases they could prevent if used proactively in an endemic setting (*X*_min_). Using a minimax strategy for each stockpile request, if the OCV doses would theoretically prevent > *X*_min_ cases, then the request should be granted; however, if the request would prevent < *X*_min_ cases, the request should be denied. If additional requests are received and doses still remain in the stockpile the same comparison with *X*_min_ is made for each request until the end of the time frame is reached and unused doses are allocated to the endemic setting. Figure 1 demonstrates that the minimax strategy is not a globally optimum strategy, because later requests may have a higher utility than earlier requests that are allocated vaccine using the minimax strategy. However, a globally optimal strategy would require estimating the probability distributions of the occurrence, size and timing of future vaccine requests and is beyond the scope of this analysis.

The minimax strategy for OCV allocation can be further refined by optimizing the number of doses allocated to each request rather than making an all-or-nothing decision. Owing to transmission dynamics and herd immunity, the per dose utility does not remain constant as vaccine coverage increases [15]. The minimax strategy for each reactive vaccination request will be to allocate OCV doses up to the amount where the marginal utility in the reactive setting of the remaining doses drops below *X*_min_ for those doses. This optimum will depend on the coverage levels in both the reactive and endemic settings. We attempt to frame our analysis in terms of the information available at the time of a request for OCV to perform a reactive campaign: the rate of epidemic growth, the time since the beginning of the epidemic, the amount of OCV in the stockpile and what endemic populations could potentially receive unused vaccine. Using mathematical models, we explore how campaign timing and the percentage of the population that can be vaccinated determine the optimal balance between reactive and endemic use of OCV, thereby identifying the strategy that minimizes the maximum number of vaccine-preventable cholera cases (i.e. the minimax strategy for responding to the request).

## 2. Methods

Cholera dynamics in both endemic and epidemic settings were represented using a dynamic, environmentally driven, susceptible–infectious–recovered (SIRB) model, where *B* represents the concentration of *Vibrio cholerae* in an environmental reservoir (electronic supplementary materials, Methods). Transmission of cholera from the environmental reservoir (*B*) to susceptible individuals (*S*) occurs at a rate *β* that depends on both the concentration of *V. cholerae* in the environmental reservoir (vibrios per ml) and the concentration (*κ*) at which an individual has a 50% chance of infection [16]. Infectious individuals excrete vibrios into the environmental reservoir at a rate 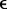 and vibrios in the environmental reservoir are lost at a rate *δ_t_*. Seasonality is included in both endemic and epidemic settings by varying the vibrio decay rate (*δ_t_*) via a sinusoidal function with varying amplitude (*σ*).

## 3. Endemic versus epidemic settings

We assume that the epidemic and endemic settings are identical (table 1) except for (i) the level of naturally acquired immunity in the population, (ii) the survival or environmental decay rate (*δ_t_*) of *V. cholerae* and (iii) the strength of seasonal forcing of *δ_t_*.

**Table 1.** Parameter values in both epidemic and endemic settings. Italicized parameter values differ between the endemic and epidemic setting.

symbol	description	endemic value	epidemic value	references
*b*	human birth and death rates (d^−1^)	6.8 × 10^−5^	6.8 × 10^−5^	—
*β*	transmission rate (rate of exposure)	0.06–0.17	0.06–0.17	[16,17]
*γ*	recovery rate (d^−1^)	0.2	0.2	[17]
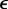	*Vibrio* excretion rate (cells ml d^−1^)	10	10	[16]
*κ*	concentration with 50% chance of infection (cells ml^−1^)	10^6^	10^6^	[16]
*δ*	Vibrio *decay rate in environment* (*d^−1^*)	*0.067*	*0.033–0.33*	[18–20]
*σ*	*seasonal amplitude in *δ*_t_*	*0.4*	*0.95*	—
*ω*_N_	loss of natural immunity (d^−1^)	0.00027	0.00027	[21]
*τ*	vaccine efficacy	0.75	0.75	[22–25]
*ω*_V_	loss of vaccine-derived immunity (d^−1^)	0.00055	0.00055	[23,26]

We assume that, in the epidemic setting, there is little or no naturally acquired immunity to cholera [27,28] and therefore, the entire population is susceptible to infection at the start of the epidemic. In highly endemic areas, the population will have had prior exposure to *V. cholerae* and at least some immunity to reinfection [21,27]. The level of natural immunity in the endemic population is simulated by running the model until cholera dynamics reach a stable equilibrium (more than 150 years) with seasonal fluctuations (annual incidence varies by less than 1%).

Another possible distinction between epidemic and endemic settings is the ability of *V. cholerae* to persist in the environment. Endemic settings may have slower decay rates for *V. cholerae* in the environment, or even positive growth during some portions of the year, which would facilitate the long-term persistence of cholera even when conditions were not favourable for transmission to humans [29,30]. We assume that 
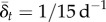
 in the endemic setting, but varies from 1/30 to 1/3 d^−1^ in the epidemic setting, because knowledge about the environmental survival of vibrios in epidemic settings is lacking [18–20]. The amplitude of seasonal variation in *δ_t_* may also differ in endemic versus epidemic settings, because a lower amplitude would favour long-term persistence, whereas larger fluctuations would favour outbreaks when conditions are favourable, but increase the odds of local extinction during the off-season. In the endemic setting, the amplitude of seasonal fluctuation was set to *σ* = 0.4, so that *δ_t_* varies from a minimum of 0.040 d^−1^ to a maximum of 0.093 d^−1^, which corresponds to a seasonal range in residence time of 10.7–25 days. In the epidemic setting *σ* = 0.95, so that *δ_t_* varies between 0.0033 and 0.129 d^−1^ when 
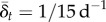
, which corresponds to a seasonal range in residence time from 7.7 to 299.9 days.

To simulate a broad range of endemic incidence levels we varied *R*_0_ from 1.05 to 2.55 by varying the value of the transmission rate *β*. In the epidemic setting, we varied both *β* and 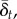 so that *R*_0_ ranged from 0.9 to 2.55, and the mean generation time (*T_C_*) varied from 8 to 35 days. The mean generation time in the endemic setting was 20 days (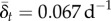). In addition to varying *R*_0_ and *T_C_*, we also varied the initial size of the epidemic population (see electronic supplementary material, Methods).

During the early stages of a cholera outbreak, it is difficult to obtain a reliable estimate of *R*_0_. *R*_0_ can be derived from estimates of the outbreak's initial rate of spread (*r*), but this requires an accurate estimate of the generation time distribution (*T_C_*) [31], and the existence of an environmental reservoir for *V. cholerae* makes it difficult to determine this distribution [17,32,33]. We estimate the initial growth rate (*r*) of the cholera epidemic after the first 30 days by assuming exponential growth, so that 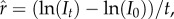 where *t* = 30 days. Each epidemic model result across the range of *R*_0_, *T_C_* and *N* was then sorted by 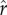 to determine whether an early estimate of the epidemic growth rate could be used to accurately predict the course of the epidemic and the optimal vaccination strategy when the values of these important epidemic variables are unknown.

## 4. Vaccination description

In the endemic setting, it is assumed that vaccination would be non-targeted and occur as part of a mass vaccination campaign completed prior to the main transmission season. A full vaccine course (two doses) is assumed to have 75% efficacy against cholera infection [22–25], and vaccine is distributed without regard to immune status. Hence, the number of susceptibles is reduced by *D*(*S*/2*N*) prior to the start of the transmission season (where *D* is the number of doses distributed).

In the epidemic setting, vaccination occurs via a reactive vaccination campaign following the start of the epidemic. We assume that all individuals receive a full course of two doses in both the endemic and epidemic settings. The epidemic cholera model (electronic supplementary material, equation S1) is modified to include vaccination (parameters described in table 1)4.1
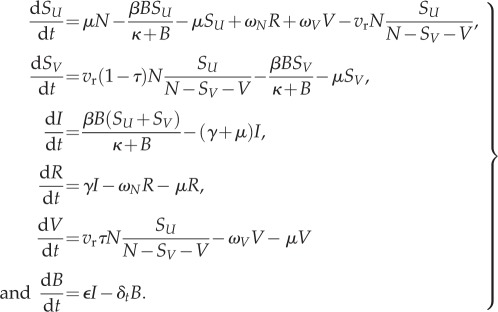


The vaccination rate *v*_r_ is the percentage of the population vaccinated per day and depends on the per cent of the population being vaccinated and the length of the reactive vaccination campaign. We assume that each individual who is vaccinated receives two doses 20 days apart and protection begins 10 days after the second dose with a probability of *τ* = 0.75 [34,35]. Vaccinated individuals move from the susceptible class (*S_U_*) to the vaccinated class (*V*) with probability *τ* or remain susceptible (*S_V_*) with probability (1 − *τ*). It is assumed that the reactive vaccination occurs over a 40 day period (20 days to administer the first round followed by 20 days for the second round of doses). In comparison, during a cholera outbreak in Guinea in 2012, 350 000 OCV doses were administered over the course of two separate campaigns that took 26 and 19 days, respectively [36]. The vaccination rate for the second round of doses is 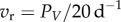, where *P_V_* is the percentage of the population receiving two doses of the vaccine. Therefore, the first vaccinated individuals will be protected starting 30 days after the start of the vaccination campaign and the last individuals will be protected 50 days after the campaign start date. We test the sensitivity of the reactive vaccination campaign to delays in campaign implementation by varying the campaign start date from 0 up to 150 days following the start of an epidemic.

We initially assume a maximum of 2 million OCV doses to be distributed across epidemic and endemic populations of 1 million each. We calculate the number of cholera infections prevented by vaccination during the first year post-vaccination by comparing the number of infections in the vaccinated population to the number of infections in an entirely unvaccinated population. The predicted outcome from a single reactive vaccination request is compared with the predicted outcome of vaccinating proactively in an endemic setting, without considering the possibility of future reactive vaccination requests. The optimal risk-averse allocation (minimax strategy) for any number of OCV doses remaining in the stockpile is determined by calculating how many cholera cases would be prevented in a reactive campaign when a fraction from 0 to 1 (in increments of 0.05) of the existing doses is allocated, with the remaining fraction used in an endemic setting. The minimax strategy is the fractional allocation that maximizes the number of infections prevented in the two populations combined. In addition to the initial conditions, we calculated the optimal allocation when there are fewer than 2 million OCV doses and also varied the size of the population at risk in the epidemic setting from 100 000 to 3 000 000.

## 5. Results

**Figure 2.**
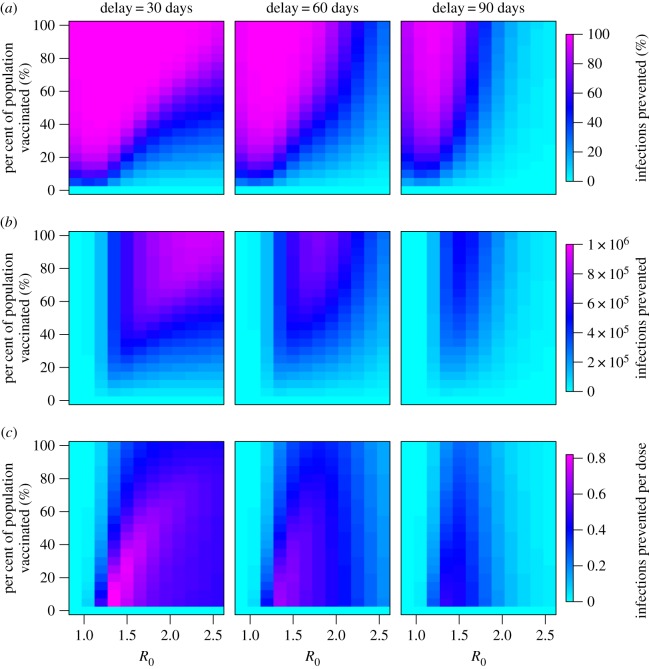
The number of cholera infections prevented via reactive vaccination in an epidemic setting as a function of *R*_0_ and the percentage of the population that is vaccinated. (*a*) Percentage of infections prevented, (*b*) total number of infections prevented and (*c*) number of infections prevented per vaccine dose are given for vaccination campaigns starting 30, 60 or 90 days after the start of the outbreak. *N* = 100 000 and 1/*δ* = 1/5 d^−1^.

A reactive vaccination campaign implemented with minimal delay can prevent a large outbreak from occurring (figure 2), but delays reduce the number of infections prevented at a given coverage level (figure 2). The wide range in effectiveness suggests that reactive vaccination campaigns that take longer to implement can still prevent a majority of cases when *R*_0_ is low or the generation time is long, but in an epidemic setting with a high *R*_0_ and short generation time the epidemic can peak before vaccination is completed (electronic supplementary material, Results).

**Figure 3.**
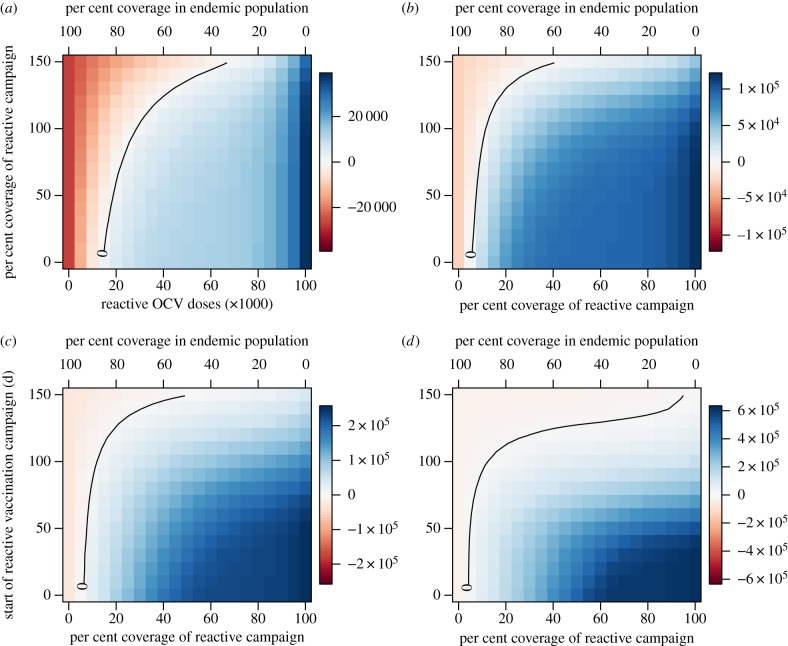
Comparison of cases prevented in the epidemic versus endemic settings as a function of the percentage of OCV doses allocated to reactive vaccination and the expected delay in reactive vaccination. As the number of OCV doses allocated to reactive vaccination increases (lower *x*-axis) the per cent coverage in an endemic population of 1 million declines accordingly (upper *x*-axis). Positive numbers (blue) indicate more cases are prevented in the epidemic setting while negative numbers (red) indicate that more cases are prevented in the endemic setting. Scenarios assume a stockpile of 2 million doses and an epidemic population at risk of 1 million. Epidemic growth rates of (*a*) 0.047, (*b*) 0.054, (*c*) 0.081 and (*d*) 0.111 d^−1^.

**Figure 4.**
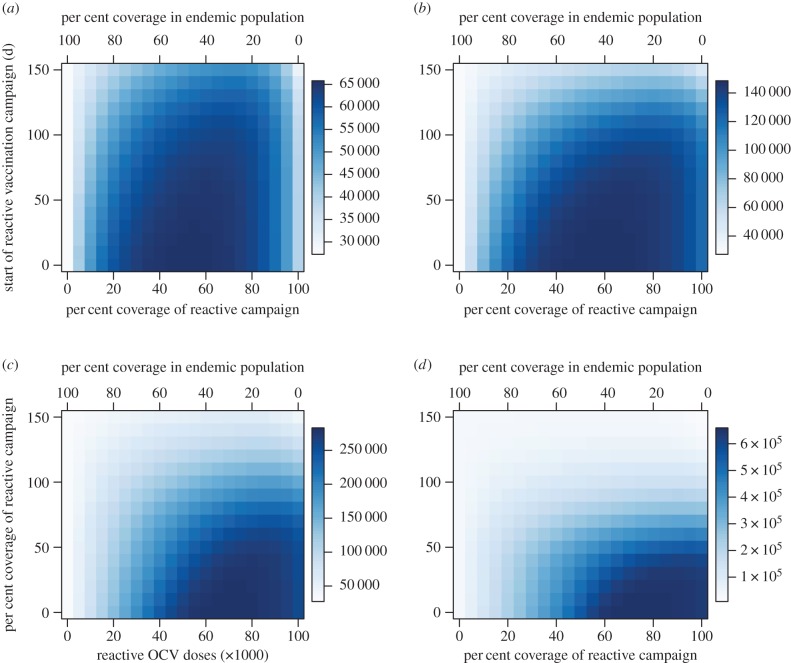
Total number of cases prevented between the epidemic and endemic settings as a function of the percentage of OCV doses allocated to reactive vaccination and the expected delay in reactive vaccination. Scenarios assume a stockpile of 2 million doses and an epidemic population at risk of 1 million. Epidemic growth rates for (*a–d*) same as in previous figure.

Across a broad range of reactive vaccination requests considered in our simulations (varying in epidemic growth rate, size of the population at risk, timing of request and number of remaining doses in stockpile), the majority of OCV doses should be allocated to reactive vaccination in epidemic settings where incidence is typically higher and more cases and deaths can be averted per dose. The number of cases prevented by reactive vaccination in the epidemic setting begins to exceed the number of cases prevented in the endemic setting at a relatively low coverage level, even when the remaining doses would provide fairly high levels of coverage in an endemic population (figure 3). The exception to this trend is when the start of reactive vaccination is delayed more than 100 days for an epidemic with a high initial growth rate (indicative of a high *R*_0_ and/or short generation time; figure 3*d*). The total cases prevented in both the epidemic and endemic settings depend on the epidemic growth rate, time to the start of the reactive vaccination campaign and the number of doses allocated to each setting. As the epidemic growth rate increases, the total number of cases prevented is highest at higher allocations to the reactive campaign (figure 4). Delays in the reactive campaign always lower the number of cases prevented and their impact on the effectiveness of reactive vaccination increases as the epidemic growth rate increases (figure 4).

**Figure 5.**
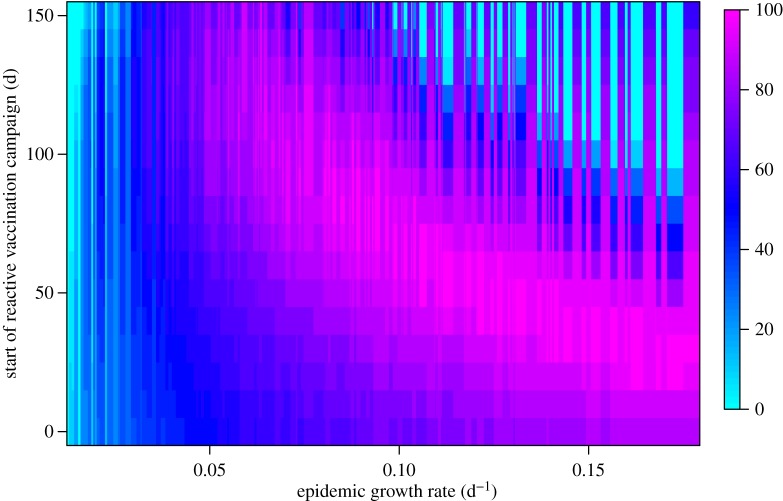
Optimal allocation (percentage of doses that maximizes the cases prevented in epidemic and endemic settings combined) of OCV doses between epidemic and endemic settings as a function of the epidemic growth rate and the expected delay to the start of the reactive vaccination campaign. Colour scale represents the optimal percentage of available doses allocated to reactive vaccination.

**Figure 6.**
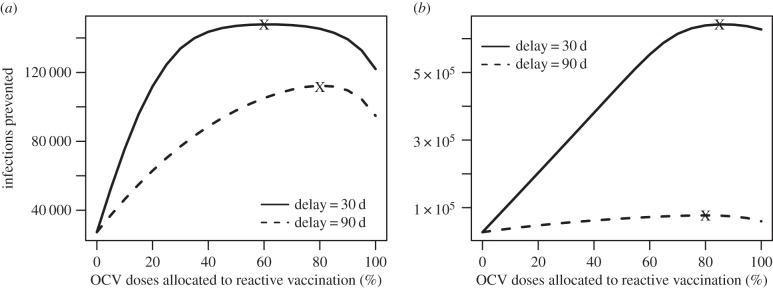
Number of cholera infections prevented when 2 million OCV doses are allocated between proactive vaccination in an endemic population of 1 million and reactively vaccinating an epidemic in a population of 1 million by per cent of OCV doses allocated to the reactive vaccination campaign in the epidemic setting. (*a*) Moderate-sized epidemic growth rate of 0.054 d^−1^ (compare with figure 3*b*) and (*b*) high epidemic growth rate of 0.111 d^−1^ (compare with figure 3*d*). *R*_0_ = 1.35 in endemic setting.

For low-to-moderate epidemic growth rates, the optimal minimax allocation of OCV doses to a reactive vaccination request increases as the delay in the campaign start date increases (figure 5). For example, for a moderate-sized epidemic in a population of 1 million (figure 3*b*), the number of cholera infections prevented is maximized by allocating 1.1 million OCV doses (60% of a 2 million dose supply) to a reactive campaign that begins within 30 days and leaving the remaining doses in the stockpile (figure 6). As the delay in the reactive campaign increases, the optimal allocation increases up to more than 80% of doses to the reactive campaign, because more doses are needed to slow an epidemic that is closer to its peak. For higher epidemic growth rates (figure 3*d*), the optimal allocation of OCV doses to the reactive setting generally decreases as the delay increases, because the epidemic is rapid enough that the majority of cases have already occurred before vaccination occurs (figures 2 and 3*d*). In general, the initial epidemic growth rate is a useful predictor of final epidemic size; however, the relationship between the initial epidemic growth rate and total incidence is not always linear, because peak transmission rates (which occur after the initial 30 day period) are dependent on the subpopulation size (electronic supplementary material, Results). The uncertainty in optimal allocation owing to this unpredictability in the final epidemic size is mainly limited to heavily delayed reactive vaccination campaigns in response to epidemics with high initial growth rates as seen in the upper right quadrant of figure 5.

**Figure 7.**
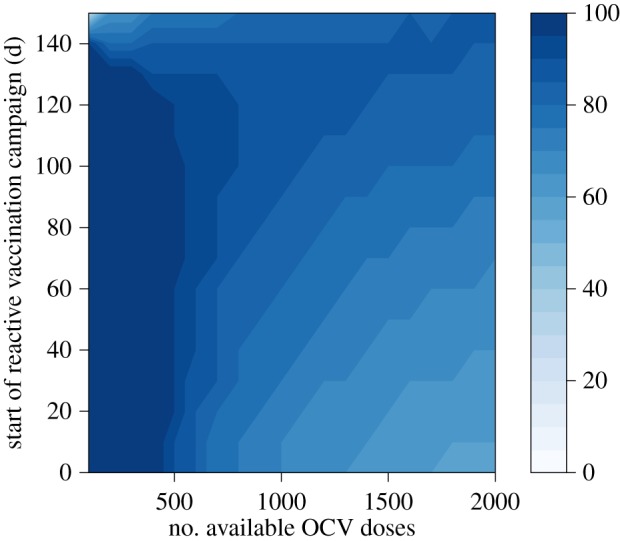
Optimal allocation of OCV doses between epidemic and endemic settings as a function of the number of doses remaining in the stockpile and the expected delay to the start of the reactive vaccination campaign. Colour scale represents the optimal percentage of available doses allocated to reactive vaccination. Epidemic growth rate of 0.054 d^−1^.

**Figure 8.**
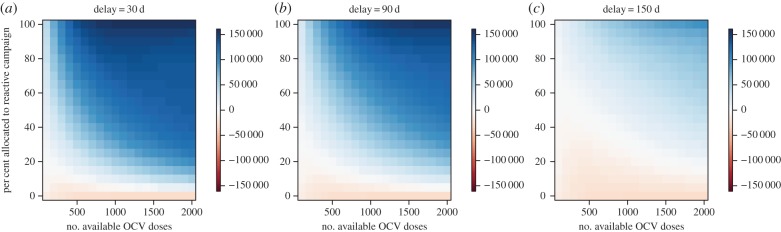
(*a*–*c*) Comparison of cases prevented in epidemic versus endemic setting as a function of the number of available OCV doses remaining in the stockpile and the percentage of these doses allocated to reactive vaccination. Positive numbers (blue) indicate more cases are prevented in the epidemic setting while negative numbers (red) indicate that more cases are prevented in the endemic setting. Epidemic growth rate of 0.054 d^−1^ (compare with figure 3*b*).

As the size of the stockpile decreases below 2 million, the optimal minimax allocation of OCV doses shifts towards the reactive setting, except for campaigns with the longest delays (figure 7). This shift occurs, because as the size of the stockpile decreases the same percentage of remaining doses yields a lower vaccination coverage level in the reactive setting and prevents fewer cases (figure 8). Therefore, a higher percentage of the remaining doses is needed to reach the vaccination coverage level in the reactive setting that still exceeds the minimum utility of the remaining doses in an endemic setting. Delays to the start of the reactive vaccination campaign also increase the allocation level necessary to reach the optimal coverage level in the reactive setting.

If the allocation criteria is maximizing the minimum number of deaths prevented rather than the minimum number of cases prevented, higher CFRs in the epidemic setting relative to the endemic setting shift the optimal minimax allocation towards reactive vaccination (electronic supplementary material, figure S16). If the number of infections prevented in an epidemic is compared with the number of infections prevented by proactive vaccination in the endemic setting over multiple years, then there is a small increase in the optimal number of OCV doses to allocate to the endemic setting for most epidemic growth rate values (electronic supplementary material, Results). The scenarios presented in figures 3–8 assume that the total population at risk in the epidemic setting is 1 million. If the population at risk is greater than 1 million, then the optimal number of OCV doses to allocate to reactive vaccination will typically be higher than for a population of 1 million (electronic supplementary material, Results).

## 6. Discussion

Owing to the limited global supply of OCV, there is the question of how to prioritize the use of the current supply, by allocating doses to reactive vaccination campaigns or proactive campaigns in highly endemic areas. The optimal risk-averse response to any reactive vaccination request is the one that allocates the remaining doses in the stockpile between the requested epidemic response and endemic use in order to maximize the minimum number of cases prevented overall. Under this minimax strategy, our results indicate that the optimal allocation of vaccine doses between endemic and epidemic settings depends mainly on five factors: (i) the expected size of the epidemic, (ii) the amount of time it will take to begin the reactive vaccination campaign in the epidemic setting, (iii) the estimated size of the population at risk of infection in the epidemic setting, (iv) the number of doses remaining in the stockpile and (v) the time horizon over which we compare performance. Because epidemic incidence rates can be much higher than incidence rates in endemic regions, there is a broad range of expected epidemic sizes where the strategy that prevents the most cholera cases (or deaths) is to allocate a majority of OCV doses to the reactive campaigns. This finding holds as long as the reactive vaccination campaigns can be implemented within the first few months of an epidemic.

In general, we found that as the expected size of an epidemic increased, the optimal allocation of OCV doses to the reactive vaccination campaign also increased. Several recent cholera epidemics have had attack rates above 1% [8,9,12,37], which is higher than the annual incidence rates typically observed in endemic settings [1]. The higher incidence in epidemic settings favours allocating a majority of the OCV supply towards reactive vaccination campaigns. This finding is dependent on the timing of the reactive vaccination campaign relative to the start and peak of the epidemic. For the parameter ranges considered, we found that as long as reactive vaccination starts within 50 days of the beginning of the epidemic the relationship between epidemic size and the amount of optimal OCV allocated to reactive vaccination was positive.

For epidemics with low-to-moderate initial growth rates (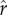) and *R*_0_ values, the timing of the reactive vaccination campaign is not as critical as it is for epidemics with higher *R*_0_ and 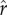 values. Our results suggest that for epidemics with moderate initial growth rates, fewer OCV doses can be allocated to the epidemic setting and can instead be saved for use in endemic settings (or saved for potential epidemics elsewhere) if the reactive vaccination can be implemented quickly. For example, for an epidemic similar in size to that observed in Zimbabwe in 2008–2009 [8], the optimal allocation to the epidemic is enough doses to cover 60–65% of the population if reactive vaccination starts less than 30 days after the epidemic begins, but more than 70% if vaccination does not start for 60 days. Even with the larger allocation required for the delayed campaign, the total number of infections prevented across the epidemic and endemic settings will be lower than a smaller allocation to an earlier reactive vaccination campaign.

For much larger and faster spreading epidemics, such as the 2010–2011 outbreak in Haiti, the timing of the reactive vaccination campaign is more critical. Unless the reactive vaccination campaign begins quickly, the majority of cases may have already occurred and it is too late to substantially slow transmission. For a population hit by an epidemic of comparable size to that in the Artibonite Department of Haiti [9], all OCV doses in the stockpile should be allocated to the reactive campaign unless the campaign starts more than 80 days after the beginning of the epidemic, in that case, less than 100% should be allocated, because the peak of the epidemic will have occurred by the time vaccination is complete. However, even as the optimal allocation to the epidemic setting declines, it still remains above 50% for at least five months, because the incidence rate post-peak remains higher than that observed in a typical endemic setting.

Our results provide some guidelines for allocating a limited OCV supply based on the expected size of an epidemic, but there is a large degree of uncertainty surrounding both the number and size of expected cholera outbreaks in a given year. The plan for the current stockpile is to distribute unused doses to endemic countries for use in proactive campaigns, which lessens this problem, because the number of cases that could be prevented by these proactive campaigns sets a minimum utility for the stockpile. If the probability of future requests were known, we might be able to determine the globally optimal percentage of vaccine to hold in reserve, but no optimal strategy will hold less vaccine in reserve than recommended by our analysis using a minimax strategy. As the size of the OCV supply diminishes, our analysis indicates that the optimal risk-averse allocation to a reactive vaccination request increases, because the cases prevented per OCV dose is highest at fairly low vaccination coverage levels. As each request is received, the minimax strategy compares only the utility of fulfilling the current request to proactive use in an endemic population, but a globally optimal strategy would also take into account the increased utility of holding at least some OCV doses in reserve for future reactive requests. If the global OCV supply increases from 2 million doses to 5–20 million doses over the next decade as planned, the percentage of the stockpile that would be allocated to each individual reactive vaccine request under a minimax would decrease, leaving more doses in reserve for any future reactive requests. This should lead to the optimal risk-averse allocation approaching a global optimum as the size of the stockpile increases. However, it will become more important to estimate the likely reactive allocation required in the near future, so that excess can be more rapidly deployed for use in endemic settings.

The optimal allocation of OCV also depends on whether the cases prevented in the endemic setting beyond a single year are considered. Because the current OCVs do provide protection for more than a year in endemic settings [23,38], a proactive vaccination campaign can reduce incidence for several years. Incorporating these additional reductions increases the proportion of the OCV supply that should be supplied to endemic settings by 5–20% for most estimated epidemic sizes (electronic supplementary material, Results). This altered allocation holds if we consider a time horizon of 2–5 years in the endemic setting (only 2–3 years in endemic areas with incidence rates above five per 1000). In our simulations, after 5 years, the loss of immunity and the build-up of susceptibles can lead to an outbreak in the endemic setting that results in as many cases as were prevented by vaccination in the previous years, suggesting utility depends on the time horizon of interest and future action in the endemic setting (e.g. future re-vaccination).

One issue with using our model to estimate the optimal OCV allocation is that epidemics with similar initial growth rates (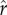) may have very different final incidence rates owing to the nonlinear relationship between the environmental *V. cholerae* concentration and transmission rates. However, these differences only become significant as the concentrations reach a significant fraction of the concentration at which individuals have a 50% chance of infection (*κ*), so they do not influence the initial growth rate. Although this can lead to a large uncertainty in the final epidemic size, this difference is only important for very large and fast spreading epidemics, such as the ones that began in Peru in 1991 or Haiti in 2010. For epidemics of this size, the optimal strategy is to allocate 100% of the available OCV doses to the reactive campaign as long as it begins relatively quickly. The largest uncertainty occurs when deciding how many doses to allocate if the campaign begins several months after the start of the epidemic. However, in this situation, the delay should also provide the opportunity to obtain additional information about the epidemic dynamics beyond the initial growth rate.

Another important consideration is the prevention of cholera deaths. Case fatality ratios differ by geographical region, country, cholera strain and locality due partially to the availability and quality of treatment [10,39]. These risk factors may lead to higher CFRs in epidemic settings where the proper surveillance and treatment infrastructure is lacking. If minimizing the number of deaths rather than cases is the primary goal, then the optimal vaccine allocation would likely shift even more towards reactive vaccination.

One final caveat is that the distinction between epidemic and endemic settings is usually not as clear-cut as presented here. Most countries classified as endemic experience significant fluctuations in incidence from year-to-year and some countries exhibit multi-year cycles that include frequent years with no or few reported cases [40]. The effects of proactive vaccination in these endemic setting will be more variable and could exceed the benefits presented here if vaccination prevents the occurrence of a periodic outbreak. In addition to reactively vaccinating in response to epidemics in previously cholera-free areas, reactive vaccination will also be considered for endemic countries that experience large seasonal outbreaks. The populations at risk in these scenarios may differ from the completely susceptible populations modelled here. The stockpile has also recently been used pre-emptively in response to humanitarian emergencies where there is a high risk of a cholera outbreak, including the delivery of 580 000 doses to refugee camps and surrounding communities in South Sudan and Ethiopia in 2013–2014 [41]. This pre-emptive use is outside the scope of our analysis because it would require estimating outbreak probabilities under a variety of humanitarian crisis scenarios. An improved understanding of the underlying risk factors and susceptibility in a variety of settings could help inform the OCV stockpile allocation process and lead to better predictions of reactive vaccination in these different settings.

The global cholera stockpile is an invaluable resource in preventing morbidity and mortality. Our analysis shows that the current focus on reactive campaigns is appropriate, and that, in general, fulfilling timely requests will minimize the potential for vaccine to be deployed in a suboptimal setting. Hopefully, supplies of OCV will become less constrained over the coming years. As they do, strategic analyses such as these should be extended to take a longer view of controlling endemic transmission and completely eliminating the risk from cholera in epidemic prone areas. Such strategic modelling efforts should be paired with efforts to improve the empirical evidence on which they are based to ensure the most rational and cost effective use of this effective vaccine.

## Supplementary Material

Supplementary Materials
