# Supplementary material for: Optimal allocation of the limited oral cholera vaccine supply between endemic and epidemic settings
Source: J R Soc Interface. 2015 Oct 6;12(111):20150703. doi: 10.1098/rsif.2015.0703 (PMC4614506; doi:10.1098/rsif.2015.0703)
Supplement: Supplementary Materials [file rsif20150703supp1.pdf]

Optimal allocation of the limited oral cholera vaccine supply between endemic and epidemic settings

Sean M. Moore* and Justin Lessler

Department of Epidemiology,
Johns Hopkins Bloomberg School of Public Health,
Baltimore, MD 21205

smoore62@jhu.edu
justin@jhu.edu

April 30, 2015

Supplementary Methods

Cholera dynamics in both endemic and epidemic settings were represented using a dynamic, environmentally-driven, susceptible-infectious-recovered (SIRB) model, where B represents the concentration of *V. cholerae* in an environmental reservoir [1],

$$\begin{aligned}
\frac{dS}{dt} &= \mu N - \frac{\beta B S}{\kappa + B} - \mu S + \omega_N R \\
\frac{dI}{dt} &= \frac{\beta B S}{\kappa + B} - (\gamma + \mu) I \\
\frac{dR}{dt} &= \gamma I - \omega_N R - \mu R \\
\frac{dB}{dt} &= \epsilon I - \delta_t B.
\end{aligned} \tag{1}$$

Individuals are born and die at an average rate, μ, into a population with a constant size, N. Transmission of cholera from the environmental reservoir (B) to susceptible individuals (S) occurs at a rate β that depends on both the concentration of *V. cholerae* in the environmental reservoir (vibrios per mL) and the concentration (κ) at which an individual has a 50% chance of infection. Recovery from infection occurs at a rate γ, and infectious individuals excrete vibrios into the environmental reservoir at a rate ϵ. Vibrios in the environmental reservoir are lost at a rate δ_t. Seasonality is included in both endemic and epidemic settings by varying the vibrio decay rate (δ_t) via a sinusodial function with varying amplitude (σ).

The basic reproductive number for the model, R_0, is,

$$R_0 = \frac{\beta \epsilon}{\delta_t \kappa (\gamma + \mu)} N. \tag{2}$$

The mean generation time or serial interval between infections (T_C) is determined by the recovery rate of infectious individuals (γ) and the decay rate of *V. cholerae* in the environment (δ_t) such that $T_C = \frac{1}{\gamma} + \frac{1}{\delta_t}$.

The force of infection (λ) experienced by a susceptible individual is determined by both the transmission rate (β) and the concentration of vibrios in the environment relative to κ,

$$\lambda = \beta \frac{B}{\kappa + B}. \tag{3}$$

The total population size was set to one million in the epidemic setting, but was then divided into individual subpopulations (N) of either 10,000, 50,000, 100,000, 200,000, 500,000, or 1,000,000. The parameters were identical in each individual subpopulation, but each subpopulation had its own water source and environmental reservoir (B) without mixing between subpopulations. The force of infection (λ) scales non-linearly with the concentration of vibrios in the environmental reservoir (B) due to the logistic equation that determines the dose-dependent infection rate (Equation 3). Therefore, a ten-fold increase in B may lead to less than a ten-fold increase in λ, with saturation becoming more pronounced as B approaches or exceeds κ. As a consequence, subpopulations of different sizes will experience different forces of infection even if all parameter values are identical. For the endemic setting varying the size of the subpopulations did not significantly alter annual incidence rates, so results for $N = 100,000$ are presented.

Supplementary Results

Proactive vaccination

Our simulations produce yearly incidence rates in an endemic setting ranging from 9.9 infections per 1000 population at $R_0 = 1.05$ to 59.7 infections per 1000 population at an $R_0 = 2.55$. If we assume that only 10% of infections are reported as suspected cases then disease incidence rates observed in the endemic setting would range from 0.9 to 6.0 per 1000. Vaccinating 10% of the endemic population reduces the incidence in the following year by 46.3–54.8% and vaccinating 50% of the population lowers incidence by 96.9–97.5%,

with higher reduction levels achieved for lower R_0 values.

If 100% of the endemic population is vaccinated the annual incidence of infection in the year following vaccination drops to 0.02 per 1000 population at $R_0 = 1.05$ to a high of 0.10 infections per 1000 population at an $R_0 = 2.55$, a reduction of over 99% across the range of R_0 values examined (Fig. 1a). For values of $R_0 < 1.5$, vaccinating 100% of the population is enough to eliminate cholera locally. For values of $R_0 > 1.5$, the annual incidence remains below 0.1 (/1000) for at least 5 years, but does begin to increase after 5 years as both natural and vaccine-derived immunity wane. If 50% of the endemic population is vaccinated the annual incidence in the following year drops to 0.25 to 1.9 per 1000, a reduction of 96.9–97.5% (Fig. 1b). Even vaccinating only 10% of the population reduces the incidence in the following year by 46.3% to 54.8%, with higher reduction levels achieved for lower R_0 values. The percentage reduction in incidence in the first year following vaccination is fairly similar across the range of R_0 values examined, but the incidence rate returns to pre-vaccination levels faster as R_0 increases (Fig. 1a). Vaccination levels that aren't adequate to eliminate cholera will eventually lead to incidence rates that exceed the long-term endemic equilibrium for a year or two (before returning to equilibrium) as susceptibles build up in the population.

Reactive vaccination

To determine what range of parameter space cholera epidemics are likely to occur in we looked at the estimates from two recent epidemics. Parameter values for δ_t and N were selected to match the estimated infection rates and R_0 values calculated by [2, 3] for Artibonite province in Haiti during 2010-2011, and Mashonaland West province, Zimbabwe during the 2008-2009 epidemic. With an incidence rate of 17.5 per 1000 and an estimated $R_0 = 1.87$, Mashonaland West province represents a moderate-to-large outbreak, while the incidence rates of 50 per 1000 up to over 250 per 1000 in some areas of Artibonite represent a very high intensity epidemic [4].

Incidence rates in the epidemic setting increase as R_0 increases, and for a given R_0 the incidence rate will be higher as the generation time decreases and for smaller subpopulation sizes (Figs. 2 and 3). While the percentage of infections averted is highest for lower R_0 values because the epidemic evolves more slowly, the total number of cases averted is highest at intermediate levels of R_0 because the infection rates are high and the epidemic still evolves slowly enough to prevent a large percentage of infections (Figs. 4,5). For the highest R_0 values and shortest generation times, the number (and percentage) of infections averted drops as the epidemic peak occurs earlier (Fig. 6).

Although the total number of infections averted is maximized by vaccinating 100% of the population, the number of infections averted per vaccine dose is maximized at much lower vaccination coverage levels (Figs. 7). As R_0 increases the vaccination percentage that maximizes the number of infections averted per dose also increases if reactive vaccination begins within less than 90 days. For example, if $N = 100,000$, $\delta_t = 1/5$, and $R_0 = 1.2$, the number of infections prevented per dose is maximized when only 5% of the population is vaccinated (0.05 infections/dose), but at $R_0 = 1.5$ the number of infections prevented per dose is maximized by vaccinating 30% of the population (0.57 infections/dose) and when $R_0 = 2.1$ the number of infections prevented per dose is maximized by vaccinating 80% of the population (0.44 infections/dose). At a given vaccination coverage level the number of cases prevented per vaccine dose is highest at moderate levels of R_0 (Fig. 7).

Delays in the start of the reactive vaccination campaign reduce the number of infections prevented at a given vaccination coverage level (Figs. 4-7). For example, a reactive vaccination campaign that begins 60 days after the first infection and covers 100% of the population will prevent between <1 and 99.5% of infections compared to 48.7 and 99.9% when the campaign starts after only 30 days (Fig. 4). Reactive vaccination campaigns that take longer to implement can still prevent a majority of cases when R_0 is low and the generation time (T_C) is long, but in an epidemic setting with a high R_0 and short T_C the epidemic can peak before vaccination is completed. As the delay in the reactive vaccina-

tion start date increases, the level of vaccination coverage that maximizes the number of infections per dose also decreases.

Optimal allocation

For a moderately high infection rate of 27.3 per 1000 in the endemic setting (corresponding to an annual case incidence rate of 3–6 per 1000 if the asymptomatic rate is 80–90%) when $R_0 = 1.35$, the optimal percentage of vaccine doses to allocate to the reactive vaccination campaign ranges from 0 to 100% based on the expected size of the epidemic and the start date of the reactive vaccination campaign in the epidemic setting (Fig. 8). Varying the annual endemic infection rate from 10–60 per 1000 does not lead to any large shifts in these results, but does shift the optimal allocation slightly towards the endemic setting for low to intermediate R_0 values in the epidemic setting or when the reactive vaccination campaign starts late relative to the start of the epidemic (Fig. 9a-f).

If the number of infections prevented in the reactive setting are compared to the number of infections prevented by proactive vaccination in the endemic setting over multiple years then there is a small increase in the optimal number of OCV doses to allocate to the endemic setting for most epidemic growth rate values (Figs. 10,11).

R_0 versus epidemic growth rate

The incidence of cholera infection generally increased as the initial epidemic growth rate ($\hat{r}$) increased, but there was considerable variation in the infection rate over small changes in $\hat{r}$ (Fig. 12a) due to epidemics in different subpopulation sizes having very similar initial growth rates, but very different final incidence rates (Fig. 12b). The relationship between $\hat{r}$ and the percentage or total number of infections prevented via vaccination is very similar to the relationship between R_0 and infections prevented shown in Fig. (**??**). However, the total number of infections prevented and the number of infections prevented per vaccine dose can vary substantially with very small changes in $\hat{r}$ due to the variation in final

incidence rate with subpopulation size (Fig. 13). Despite this variability the optimal allocation for a particular value of $\hat{r}$ does not vary much if the delay is short (Fig. 14a). For longer delays the optimal allocation remains insensitive to small changes at lower $\hat{r}$ values, but becomes very uncertain for high $\hat{r}$ values (Fig. 14b,c).

Size of susceptible population

The scenarios presented in the main text assumed that the total population at risk in the epidemic setting is one million. If the population at risk is less than one million the optimal percentage of the population to vaccinate remains the same because the optimal percentage is determined by the vaccination coverage level at which the incidence in the epidemic setting drops below the incidence in the vaccinated endemic population (Fig. 15). Therefore, the number of doses to allocate to the reactive campaign scales linearly with the size of the population at risk up to one million. If the population at risk is greater than one million then the OCV supply is insufficient to cover 100% of the population at risk and the percent of the epidemic population that is vaccinated will decrease even as the optimal number of OCV doses to allocate to reactive vaccination increases. As the size of the population at risk increases above one million the optimal allocation to reactive vaccination approaches 100% of the available OCV doses (Fig. 15).

References

[1] C.T. Codeco. "Endemic and epidemic dynamics of cholera: the role of the aquatic reservoir". In: *BMC Infect. Dis.* 1.1 (2001), p. 1. DOI: 10.1186/1471-2334-1-1.

[2] Z. Mukandavire et al. "Estimating the reproductive numbers for the 2008-2009 cholera outbreaks in Zimbabwe". In: *Proc. Natl. Acad. Sci. U. S. A.* 108.21 (2011), pp. 8767–8772.

[3] Z. Mukandavire, D.L. Smith, and G.J. Morris Jr. "Cholera in Haiti: Reproductive numbers and vaccination coverage estimates". In: *Scientific Reports* 3 (2013). DOI: 10.1038/srep00997.

[4] J Gaudart et al. "Spatio-Temporal Dynamics of Cholera during the First Year of the Epidemic in Haiti". In: *PLoS Negl Trop Dis* 7.4 (2013), e2145. DOI: 10.1371/journal.pntd.0002145.

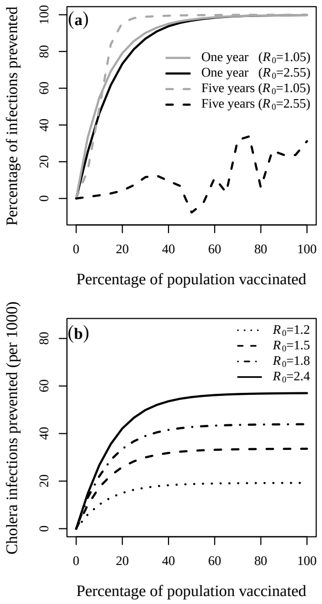

Figure 1: (a) Percentage of infections prevented by vaccination in an endemic setting after one and five years with $R_0 = 1.05$ or $R_0 = 2.55$, and (b) the number of infections prevented per 1000 population after one year at various R_0 values.

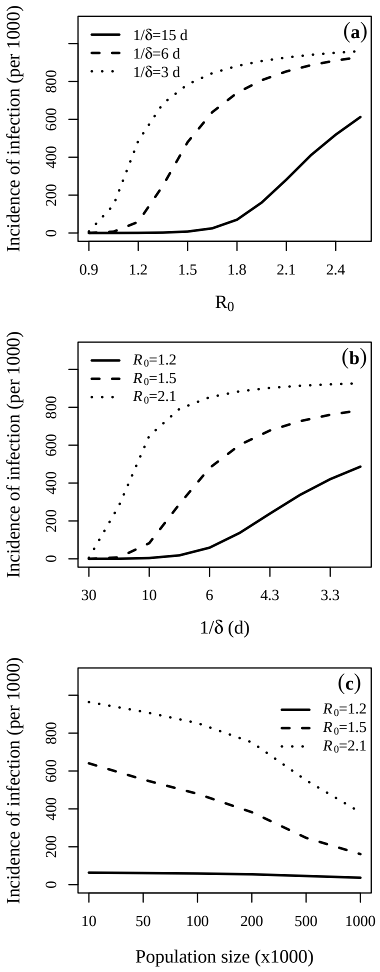

Figure 2: The incidence of infection in an epidemic setting (a) by R_0, (b) the environmental decay rate of V. cholerae (δ), and (c) population size. For (a) and (b) the population size is $N = 100,000$ and for (c) $1/\delta = 1/6d^{-1}$.

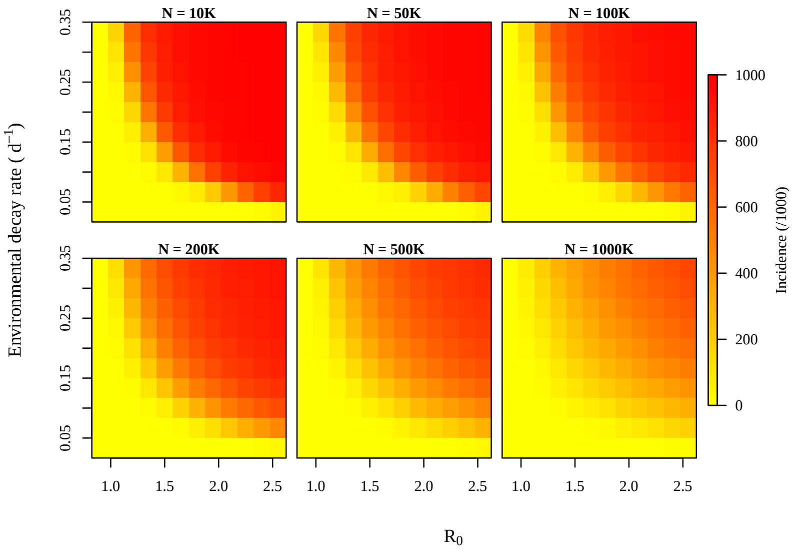

Figure 3: Incidence of cholera infections (per 1000) in an epidemic setting by R_0, the environmental decay rate of *V. cholerae* (δ), and the size of the population.

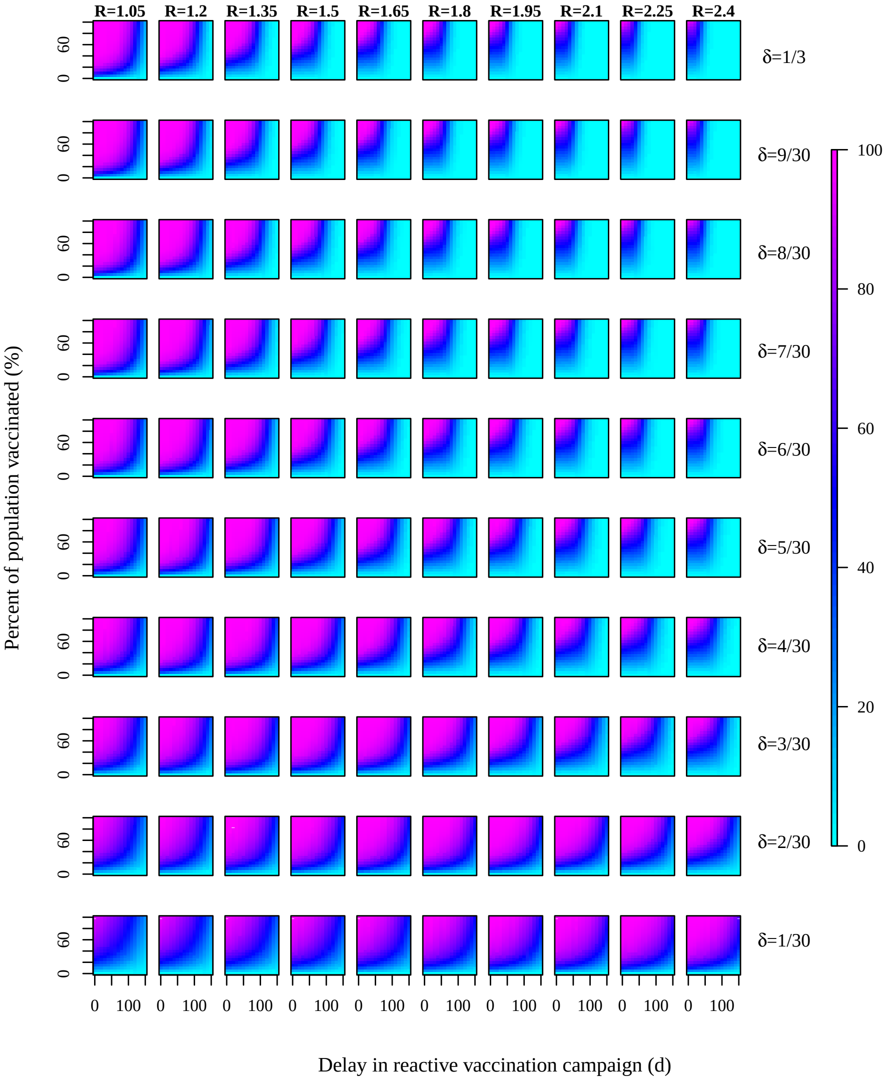

Figure 4: Percentage of cholera infections prevented by reactive vaccination campaign in an epidemic setting for a range of values of R_0 and the environmental decay rate of *V. cholerae* (δ). X-axis for each figure is delay in the start of the vaccine campaign (number of days after first infection) and Y-axis is percentage of the population that is vaccinated. N=100,000.

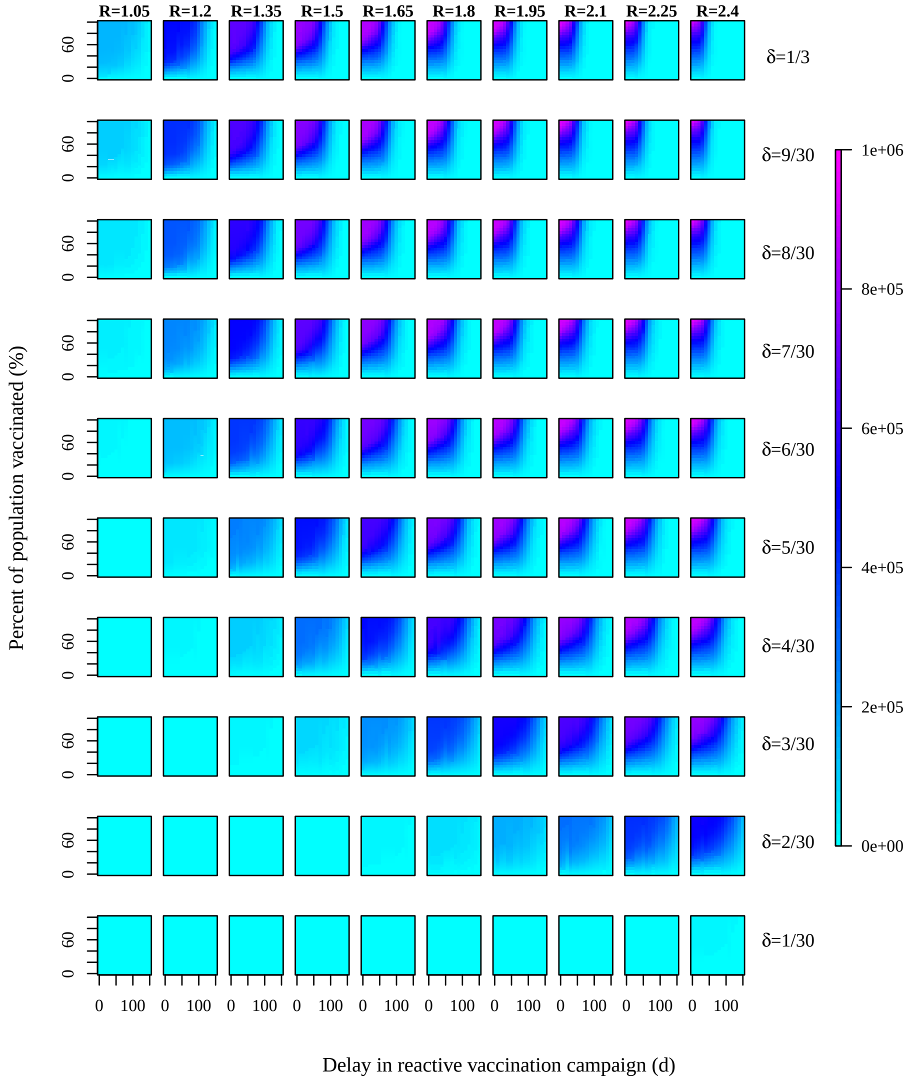

Figure 5: Total number of cholera infections prevented by reactive vaccination campaign in an epidemic setting for a range of values of R_0 and the environmental decay rate of *V. cholerae* (δ). X-axis for each figure is delay in the start of the vaccine campaign (number of days after first infection) and Y-axis is percentage of the population that is vaccinated. N=100,000.

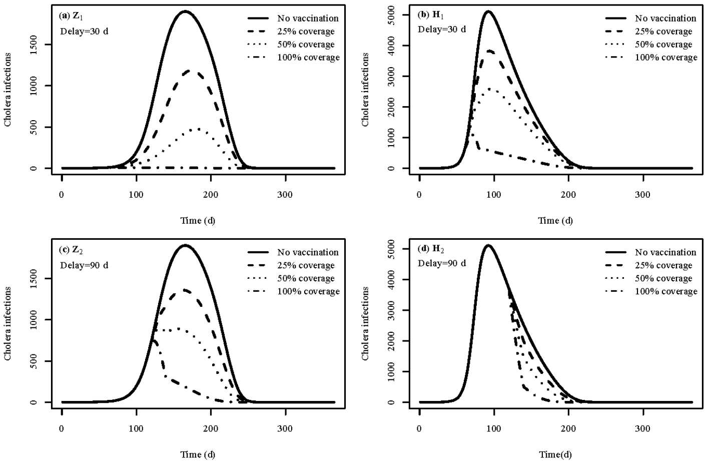

Figure 6: Epidemic curves with and without reactive vaccination 30 or 90 days after the start of an epidemic with similar R_0 and incidence values to (a-c) the 2008-2009 cholera outbreak in Mashonaland West province, Zimbabwe and (b-d) the 2010-2011 cholera outbreak in Artibonite department, Haiti.

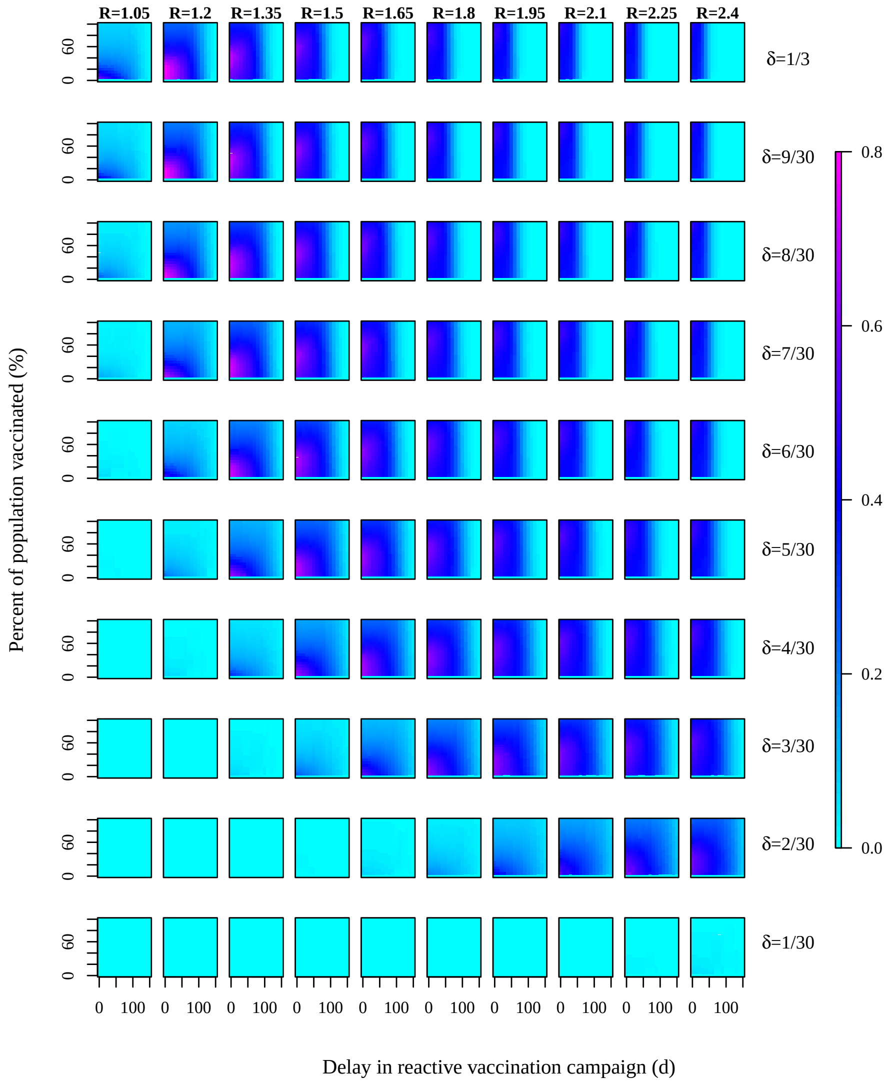

Figure 7: Number of cholera infections prevented per vaccine dose in a reactive vaccination campaign in an epidemic setting for a range of values for R_0 and the environmental decay rate of *V. cholerae* (δ). X-axis for each figure is delay in the start of the vaccine campaign (number of days after first infection) and Y-axis is percentage of the population that is vaccinated. N=100,000.

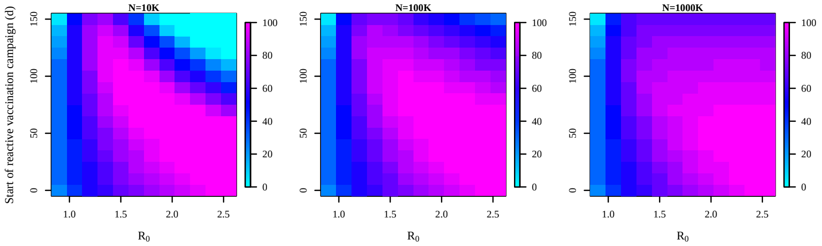

Figure 8: Optimal allocation of cholera OCV doses (represented as percentage of OCV doses to allocate to reactive vaccination in epidemic setting) between epidemic and endemic settings as a function of R_0 in the epidemic setting and the timing of the reactive vaccination campaign relative to the start of the epidemic for three different epidemic subpopulation sizes (10,000, 100,000, and 1,000,000). Color scale represents the optimal percentage of available doses allocated to reactive vaccination. $1/\delta = 1/5d^{-1}$ in epidemic setting, $R_0 = 1.35$ in endemic setting.

(a)

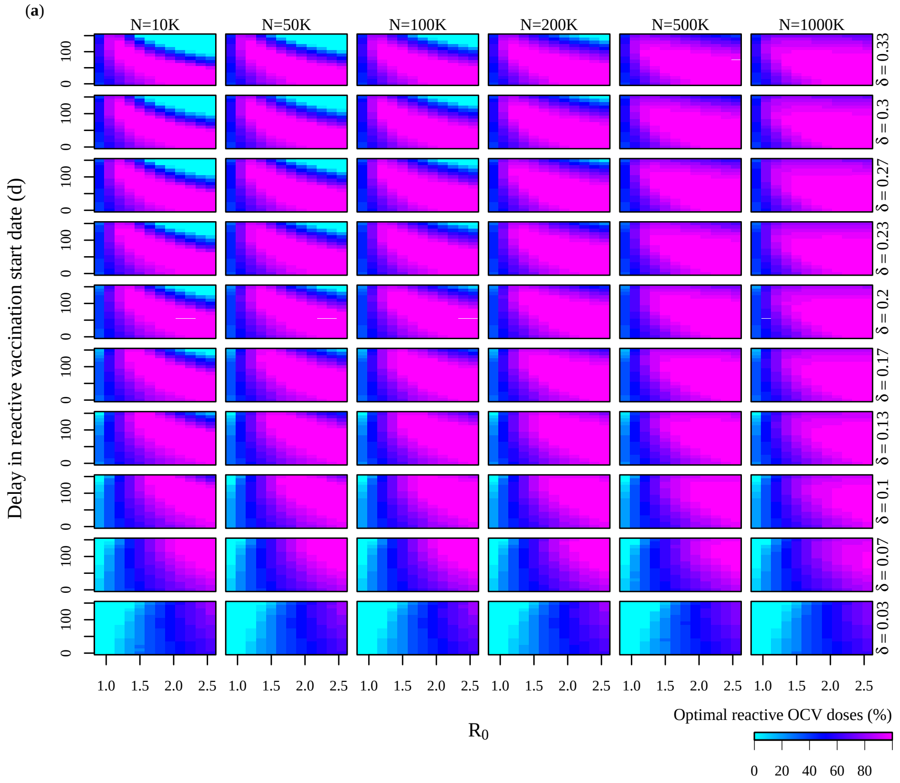

N=10K
N=50K
N=100K
N=200K
N=500K
N=1000K
δ = 0.33
δ = 0.3
δ = 0.27
δ = 0.23
δ = 0.2
δ = 0.17
δ = 0.13
δ = 0.1
δ = 0.07
δ = 0.03
Delay in reactive vaccination start date (d)
0
100
1.0
1.5
2.0
2.5
R0
Optimal reactive OCV doses (%)
0
20
40
60
80

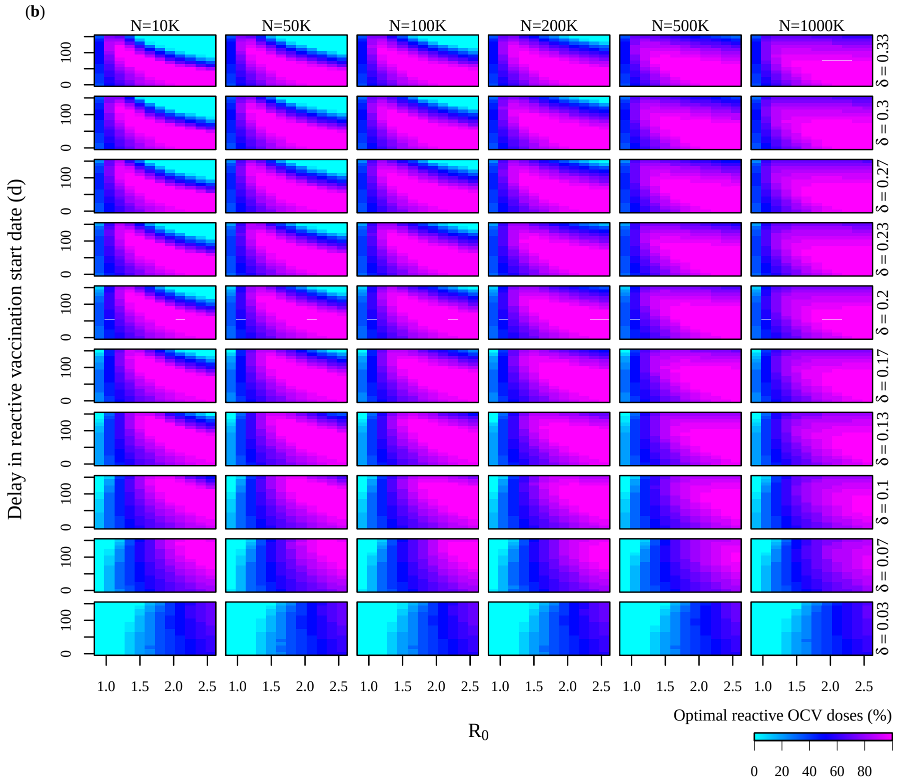
(b)
N=10K
N=50K
N=100K
N=200K
N=500K
N=1000K
Delay in reactive vaccination start date (d)
0
100
δ = 0.33
δ = 0.3
δ = 0.27
δ = 0.23
δ = 0.2
δ = 0.17
δ = 0.13
δ = 0.1
δ = 0.07
δ = 0.03
1.0 1.5 2.0 2.5
R0
Optimal reactive OCV doses (%)
0 20 40 60 80

(c)

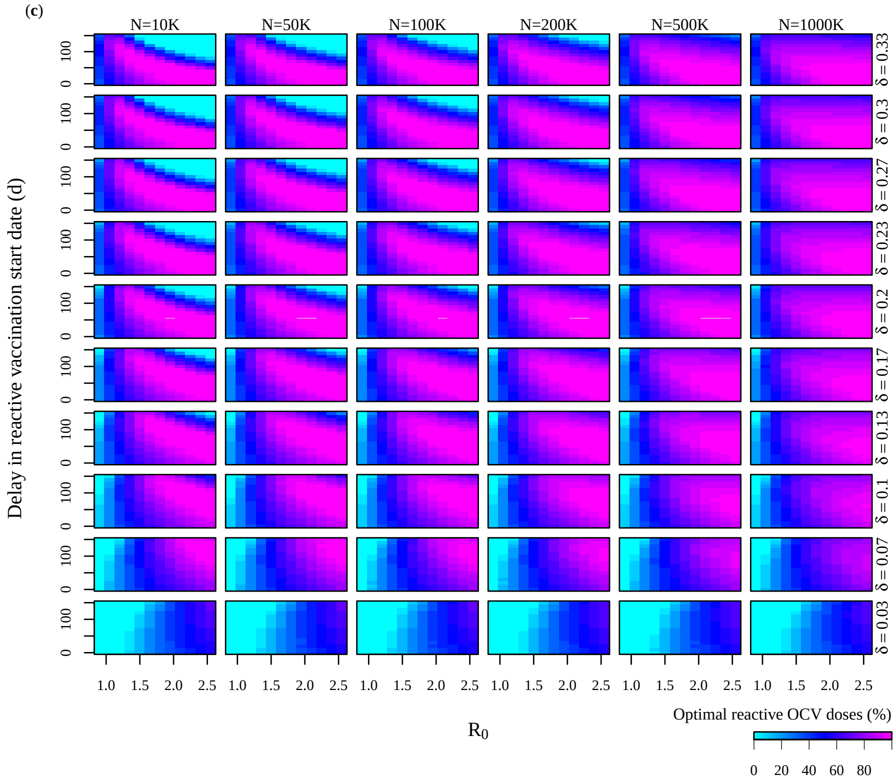
N=10K
N=50K
N=100K
N=200K
N=500K
N=1000K
δ = 0.33
δ = 0.3
δ = 0.27
δ = 0.23
δ = 0.2
δ = 0.17
δ = 0.13
δ = 0.1
δ = 0.07
δ = 0.03
Delay in reactive vaccination start date (d)
0
100
1.0
1.5
2.0
2.5
R0
Optimal reactive OCV doses (%)
0
20
40
60
80

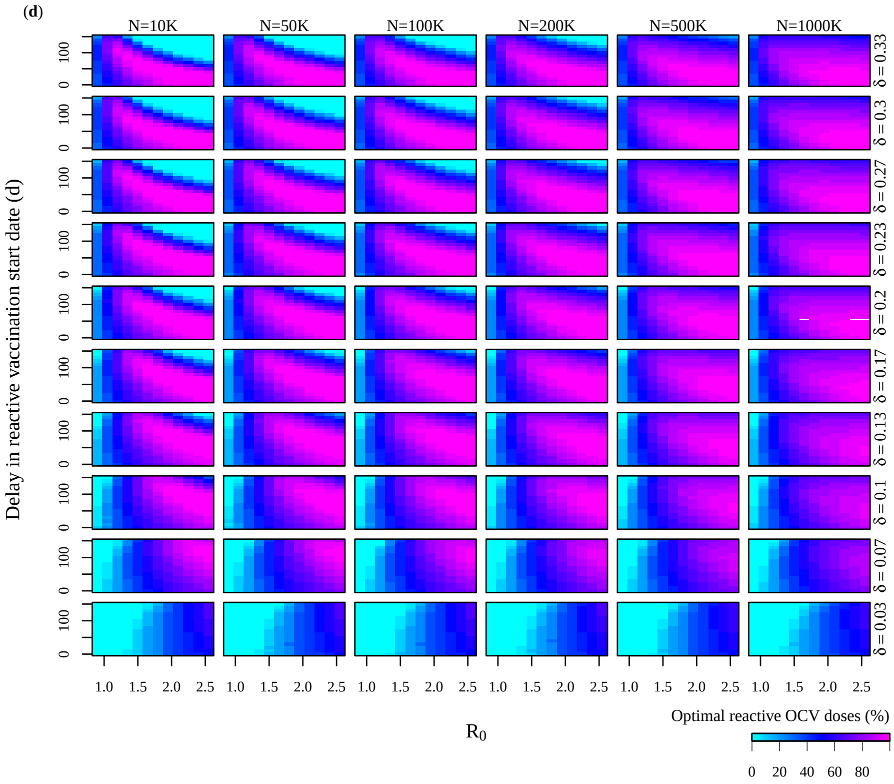
(d)
N=10K
N=50K
N=100K
N=200K
N=500K
N=1000K
δ = 0.33
δ = 0.3
δ = 0.27
δ = 0.23
δ = 0.2
δ = 0.17
δ = 0.13
δ = 0.1
δ = 0.07
δ = 0.03
Delay in reactive vaccination start date (d)
0
100
1.0
1.5
2.0
2.5
R0
Optimal reactive OCV doses (%)
0
20
40
60
80

(e)

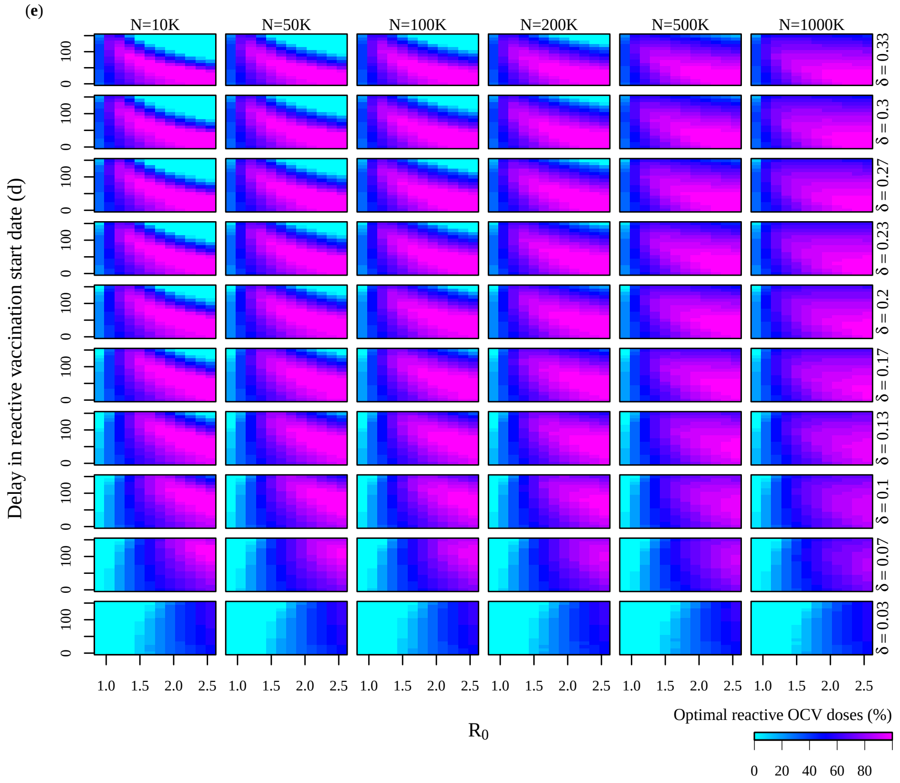
N=10K
N=50K
N=100K
N=200K
N=500K
N=1000K
Delay in reactive vaccination start date (d)
0
100
δ = 0.33
δ = 0.3
δ = 0.27
δ = 0.23
δ = 0.2
δ = 0.17
δ = 0.13
δ = 0.1
δ = 0.07
δ = 0.03
1.0 1.5 2.0 2.5
R0
Optimal reactive OCV doses (%)
0 20 40 60 80

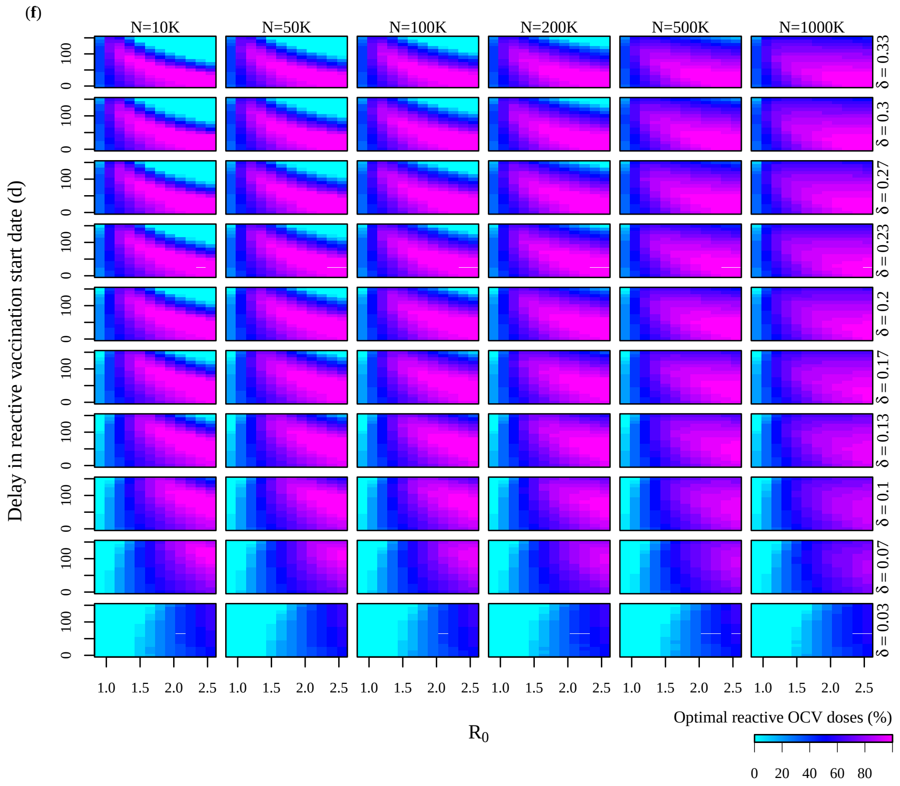

Figure 9: The allocation of cholera OCV doses that maximizes the number of cholera infection prevented in the endemic and epidemic settings combined represented as a percentage of the 2 million OCV doses that should be allocated to the reactive vaccination campaign in the epidemic setting. The value of R_0 and annual incidence of infection in the endemic setting increases from (a) to (f) as follows: (a) $R_0 = 1.05$, 9.9 infections (/1000), (b) $R_0 = 1.2$, 19.3 infections (/1000), (d) $R_0 = 1.35$, 27.3 infections (/1000), (d) $R_0 = 1.65$, 39.3 infections (/1000), (e) $R_0 = 1.95$, 51.5 infections (/1000), and (f) $R_0 = 2.55$, 59.7 infections (/1000). The x-axis for each figure is a range of $R_0 = 0.9 - 2.55$ in the epidemic setting and the y-axis is the length of the delay in the reactive vaccination campaign after the start of the epidemic (in days). a of epidemic population size $N = 10,000 - 1,000,000$ and environmental decay rate δ.

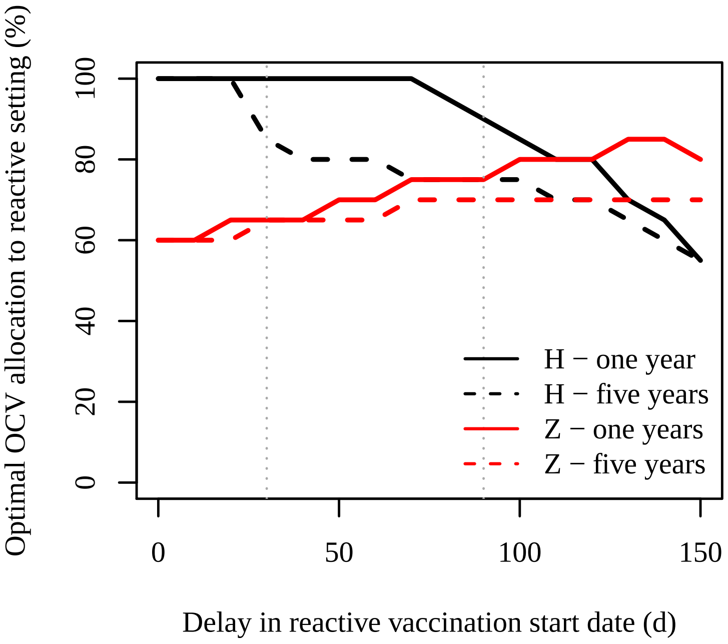

Figure 10: Optimal allocation of 2 million OCV doses to a reactive vaccination campaign versus proactive vaccination in an endemic setting as a function of the delay in the reactive vaccination campaign. Solid lines compare number of infections prevented in the epidemic setting to number of infections prevented via vaccination in the endemic setting one year post-vaccination. Dashed lines compare number of infections prevented in the epidemic setting to number of infections prevented via vaccination in the endemic setting over a five year period. Black lines are for an epidemic with R_0 and incidence values similar to the 2008-2009 cholera outbreak in Mashonaland West province, Zimbabwe and red lines are for parameter values similar to the 2010-2011 cholera outbreak in Artibonite department, Haiti. $R_0 = 1.35$ in endemic setting.

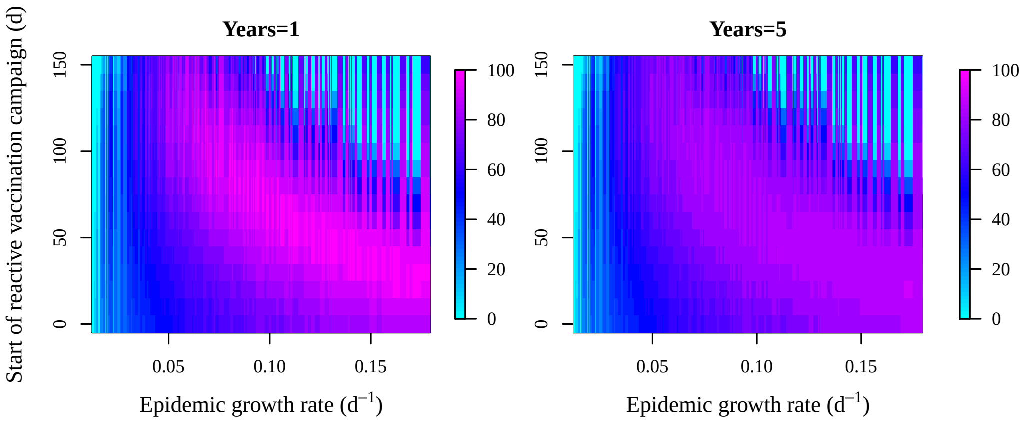

Figure 11: Optimal allocation of cholera OCV doses to the epidemic reactive vaccination campaign as a function of $\hat{r}(d^{-1})$ in the epidemic setting and the timing of the reactive vaccination campaign relative to the start of the epidemic based on maximizing the number of cholera infections prevented in the epidemic outbreak and over either (a) one or (b) five years post vaccination in the endemic setting. Color scale represents the optimal percentage of available doses allocated to reactive vaccination.

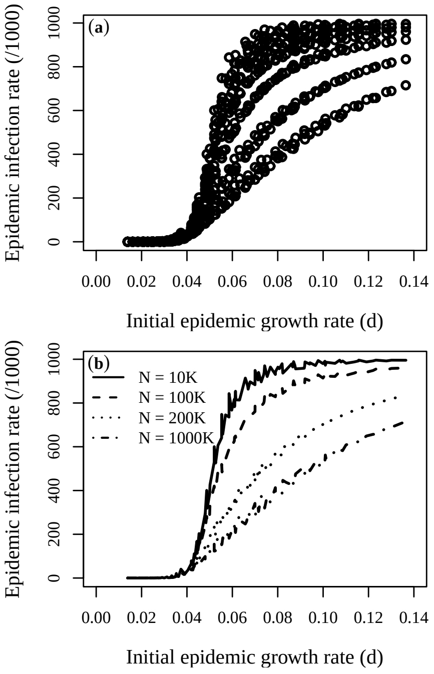

Figure 12: Number of cholera infections per 1000 population by the epidemic growth rate ($\hat{r}$) estimated at day 30 for (a) all population sizes combined and (b) population sizes of 10,000, 100,000, 200,000, and 1,000,000 shown separately. Range of R_0 is 0.9 to 2.55 and range of $1/\delta_t$ is 3 to 30 days.

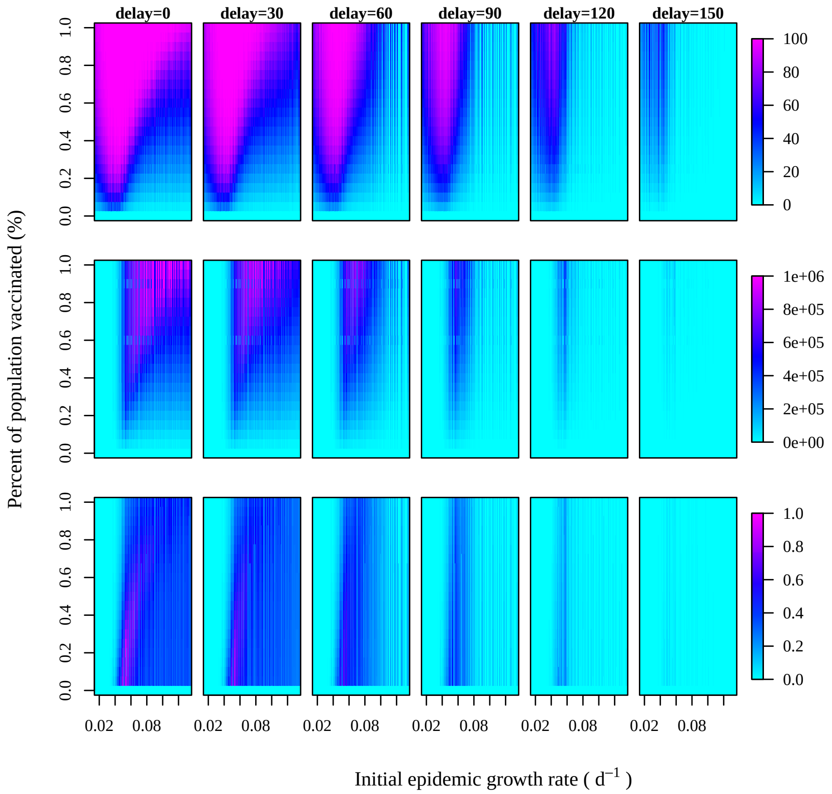

Figure 13: The number of cholera infections prevented via reactive vaccination in an epidemic setting as a function of the initial epidemic growth rate ($\hat{r}$) and the percentage of the population that is vaccinated. Percent of infections prevented, total number of infections prevented, and number of infections prevented per vaccine dose are given for vaccination campaigns starting 0, 30, 60, 90, 120, or 150 days after the start of the outbreak.

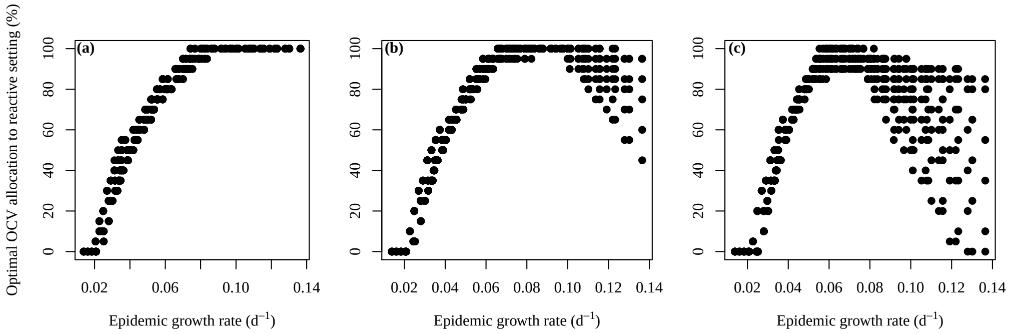

Figure 14: Optimal allocation of cholera OCV doses to the epidemic reactive vaccination campaign as a function of $\hat{r}(d^{-1})$ in the epidemic setting for reactive vaccination campaigns that start (a) 30, (b) 60, or (c) 90 days after the start of the epidemic.

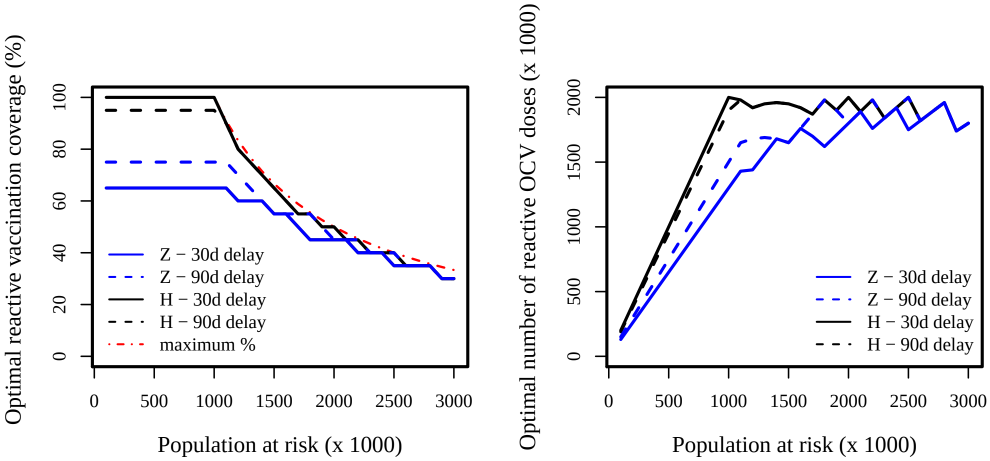

Figure 15: Optimal allocation of 2 million OCV doses to a reactive vaccination campaign versus proactive vaccination in an endemic setting as a function of the size of the population at risk in the epidemic setting. (A) Optimal percentage of the population to vaccinate in a reactive campaign and (B) the number of OCV doses to allocate to the reactive campaign. Blue lines are for an epidemic with R_0 and incidence values similar to the 2008-2009 cholera outbreak in Mashonaland West province, Zimbabwe and black lines are for parameter values similar to the 2010-2011 cholera outbreak in Artibonite department, Haiti. $R_0 = 1.35$ in endemic setting.

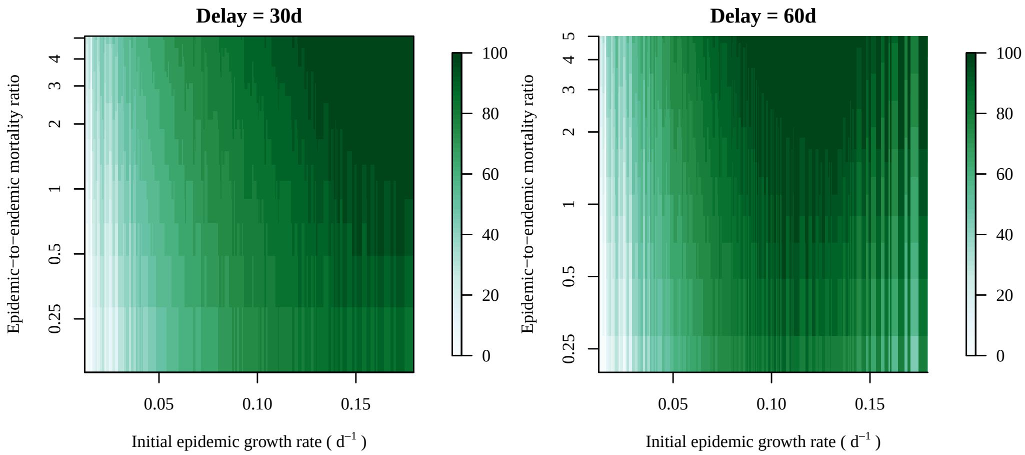

Figure 16: Optimal allocation of OCV doses (based on minimizing mortality) between epidemic and endemic settings as a function of the epidemic growth rate and the ratio of the case fatality rate in the endemic and epidemic settings. Color scale represents the optimal percentage of available doses allocated to reactive vaccination. At a ratio of 1:1 the optimal allocation is the same as the allocation based on cases prevented.
